# Cardiac magnetic resonance evidence of diffuse myocardial fibrosis in patients with mitral valve prolapse

**DOI:** 10.1186/1532-429X-17-S1-P337

**Published:** 2015-02-03

**Authors:** An H Bui, Sébastien Roujol, Murilo Foppa, Kraig V Kissinger, Beth Goddu, Thomas H Hauser, Peter J Zimetbaum, Warren J Manning, Reza Nezafat, Francesca N Delling

**Affiliations:** 1 Medicine (Division of Cardiology), Beth Israel Deaconess Medical Center, Boston, MA, USA

## Background

Diffuse myocardial fibrosis can be assessed by cardiac magnetic resonance (CMR) using the myocardial longitudinal relaxation time constant (T1). Mitral valve prolapse (MVP) is a common valvulopathy with known arrhythmic complications and in-vitro evidence of overexpression of pro-fibrotic TGF-beta. Papillary muscle fibrosis has been described in MVP, but the potential association of MVP with diffuse myocardial fibrosis is unknown. This association is important as it may increase our future understanding of ventricular arrhythmias in MVP.

## Methods

A retrospective analysis was performed on images of 41 consecutive MVP patients referred for CMR between 2006-2011. Age and gender matched controls (n = 31) free of significant cardiac disease based on clinical and CMR findings were also identified. Arrhythmia analysis was available in a subset of patients. Complex ventricular arrhythmia (ComVA) was defined as Grade III or higher by the Lown and Wolf classification on Holter or event monitor. CMR images were acquired using a Philips Achieva 1.5 T CMR scanner. Left ventricular (LV) septal T1 times were derived from Look-Locker sequences after administration of 0.2 mmol/kg gadopentetate dimeglumine. Late gadolinium enhancement (LGE) CMR images were available for all subjects. Velocity encoded CMR was used to quantify mitral regurgitation (MR) fraction (MRF).

## Results

MVP patients had significantly shorter post-contrast T1 times when compared to controls (334 ± 52 vs. 363 ± 58 ms; p = 0.03) (Figure) despite similar LV ejection fraction (61 ± 3 vs 60 ± 6 %, p = 0.84). MVP patients had greater LV end-diastolic volume and MRF compared to controls (136 ± 4.2 vs 84 ± 17 ml/m^2^, and 21 ± 24 vs 0%, both p < 0.05). There was no significant difference in post-contrast T1 times between MVP subjects with ≤ mild MR (MRF 0-15%) and > mild MR (RF > 16%) (347 vs. 334 ms; p = 0.59). Among MVP patients with available arrhythmia data (n = 19), those with ComVA (n=12) had even shorter post-contrast T1 time when compared to controls (322 ± 42 vs. 363 ± 58 ms; p = 0.016). In the MVP ComVA subgroup, the majority (11/12 or 91%) had T1 times ≤ 350 ms, but only 5/12 or 41% had papillary muscle LGE, p = 0.009.

**Figure 1:**
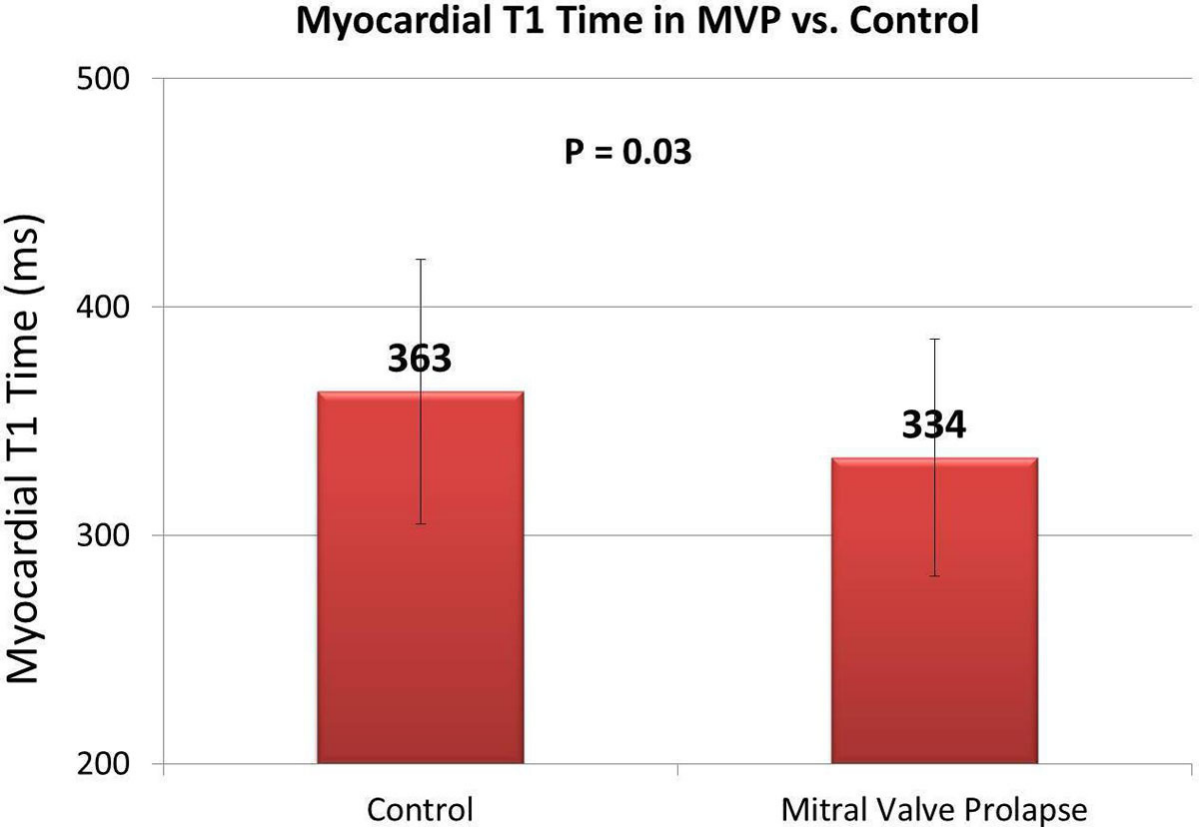
Post-contrast myocardial T_1_ time in mitral valve prolapse patients and in the control group.

## Conclusions

MVP is associated with reduced post-contrast T_1_ times despite preserved LV systolic function, suggestive of subclinical diffuse myocardial fibrosis. Mitral regurgitation alone is less likely to be the primary contributor to diffuse LV interstitial derangement in MVP. Complex ventricular arrhythmia is observed in MVP patients with diffuse myocardial fibrosis even in the absence of papillary muscle LGE. Further studies are needed to clarify if ventricular arrhythmias in MVP are secondary to diffuse rather than localized fibrosis.

## Funding

Dr Delling was supported by NIH K23HL116652.

